# Identification of Broad-Spectrum MMP Inhibitors by Virtual Screening

**DOI:** 10.3390/molecules26154553

**Published:** 2021-07-28

**Authors:** Aleix Gimeno, Doretta Cuffaro, Elisa Nuti, María José Ojeda-Montes, Raúl Beltrán-Debón, Miquel Mulero, Armando Rossello, Gerard Pujadas, Santiago Garcia-Vallvé

**Affiliations:** 1 Research Group in Cheminformatics & Nutrition, Departament de Bioquímica i Biotecnologia, Campus de Sescelades, Universitat Rovira i Virgili, 43007 Tarragona, Catalonia, Spain; aleix.givi2@gmail.com (A.G.); mjoseom88@gmail.com (M.J.O.-M.); raul.beltran@urv.cat (R.B.-D.); miquel.mulero@urv.cat (M.M.); 2 Joint IRB-BSC-CRG Program in Computational Biology, Institute for Research in Biomedicine (IRB Barcelona), The Barcelona Institute of Science and Technology, Baldiri Reixac 10-12, 08020 Barcelona, Catalonia, Spain; 3 Department of Pharmacy, University of Pisa, Via Bonanno 6, 56126 Pisa, Italy; doretta.cuffaro@farm.unipi.it (D.C.); elisa.nuti@farm.unipi.it (E.N.); armando.rossello@farm.unipi.it (A.R.); 4 Nutrigenomics Research Group, Department of Biochemistry and Biotechnology, Universitat Rovira i Virgili, 43007 Tarragona, Catalonia, Spain

**Keywords:** photoaging, skin treatment, non-selective matrix metalloproteinases, natural products, bioactivity prediction, in vitro validation

## Abstract

Matrix metalloproteinases (MMPs) are the family of proteases that are mainly responsible for degrading extracellular matrix (ECM) components. In the skin, the overexpression of MMPs as a result of ultraviolet radiation triggers an imbalance in the ECM turnover in a process called photoaging, which ultimately results in skin wrinkling and premature skin ageing. Therefore, the inhibition of different enzymes of the MMP family at a topical level could have positive implications for photoaging. Considering that the MMP catalytic region is mostly conserved across different enzymes of the MMP family, in this study we aimed to design a virtual screening (VS) workflow to identify broad-spectrum MMP inhibitors that can be used to delay the development of photoaging. Our in silico approach was validated in vitro with 20 VS hits from the Specs library that were not only structurally different from one another but also from known MMP inhibitors. In this bioactivity assay, 18 of the 20 compounds inhibit at least one of the assayed MMPs at 100 μM (with 5 of them showing around 50% inhibition in all the tested MMPs at this concentration). Finally, this VS was used to identify natural products that have the potential to act as broad-spectrum MMP inhibitors and be used as a treatment for photoaging.

## 1. Introduction

The extracellular matrix (ECM) consists of a network of macromolecules which not only provide physical support to the cell, but also transmit mechanical and molecular signals to communicate with the surrounding cells [1]. The three major components of the ECM are: (a) glycosaminoglycans, usually covalently linked to protein in the form of proteoglycans, large and highly charged polysaccharides that form a highly hydrated gel-like substance, which resists compressive forces and allows nutrients, metabolites and hormones to diffuse; (b) fibrous proteins (primarily members of the collagen family), which give the ECM both structure and elasticity; and (c) a large and varied assortment of glycoproteins, which help cells migrate, settle and differentiate in the appropriate locations. As important as the ability of cells to build and bind to the ECM is their ability to degrade it. ECM degradation is required in many cellular processes as cells may need to stretch out in order to divide, detach from other cells in order to migrate, or remove cellular material in order for the tissue to grow, be repaired, and be maintained in a continuous turnover of ECM components [1].

Matrix metalloproteinases (MMPs) are the family of proteases that are mainly responsible for degrading ECM components. These enzymes are dependent on Ca^2+^ or Zn^2+^ and degrade different components of the ECM with different specificity [1]. As MMPs are expressed in different tissues and have different substrate specificities, their uncontrolled activity, and thus the excessive degradation of different ECM components in different tissues, plays a role in a wide range of pathologies, including vascular diseases [2], inflammatory bowel disease [3,4], liver fibrosis [5], osteoarthritis [6,7] and cancer [8,9]. Therefore, MMP inhibition has been suggested as an important therapeutic tool to fight these diseases [10,11,12].

The first approach to the design of MMP inhibitors was the development of peptidomimetic inhibitors (e.g., batimastat and marimastat) that used a hydroxamic acid moiety to block the cleavage of collagen by chelating the catalytic Zn^2+^. Next, a second generation of small-molecule inhibitors was developed presenting different functional groups (i.e., hydroxamates, carboxylates, thiols and phosphorous-based) as Zn^2+^ binding groups [10]. Unfortunately, the administration of these non-selective MMP inhibitors resulted in the development of a musculoskeletal syndrome (characterized by a variety of clinical signs, including joint stiffness, inflammation and symptoms manifested as pain in the hands, arms and shoulders) and failure in clinical trials [13,14,15]. Although none of the explanations for the occurrence of the musculoskeletal syndrome proposed over the years have been confirmed, this adverse effect is believed to be a result of broad-spectrum MMP inhibition [16,17]. MMPs are also overexpressed in basal cell carcinoma, which is the most common type of human skin cancer [18] and in cutaneous squamous cell carcinoma, which is the most common metastatic skin cancer [19]. The daily oral administration of marimastat was also assayed for melanoma treatment but phase 2 assays revealed limited activity on this skin cancer [20]. Therefore, oral MMP selective inhibitors could be of interest for the treatment of the different types of skin cancer while avoiding the secondary effects of non-selective MMP inhibitors. In order to achieve such selectivity, drug discovery efforts have focused on exploiting the differences at the S1′ pocket among different MMPs [21]. Recently, a computational study demonstrated how the variability in the S1′ pocket characterizes each MMP in terms of hydrophobicity and electrostatic properties and how this variability can be rationally exploited to obtain selective MMP inhibitors (i.e., hydrophobic interactions are relevant for the selectivity of MMP-12 inhibitors; that adding a negative charge to the S1″ pocket increases the selectivity of MMP-13 inhibitors; and that the presence of a negative electrostatic environment in the S1′ pocket contributes to inhibitor selectivity over MMP-3 and MMP-8) [21].

Ultraviolet radiation has been shown to increase the expression of MMPs in the skin [22]. Overexpression increases the activity of these enzymes and ultimately increases the degradation of ECM components, which develops into skin wrinkling, a characteristic of premature skin ageing [23]. Therefore, this overexpression of MMPs has been proposed as the main mechanism behind premature skin ageing caused by the action of ultraviolet radiation (also referred to as photoaging) [24]. In this regard, compounds capable of inhibiting MMP enzymes by binding to their catalytic site and reducing their activity may restore the balance in the ECM turnover of the skin and should have beneficial effects on the treatment of photoaging.

As mentioned before, the catalytic site of MMPs is highly conserved among the various enzymes of the MMP family [12,25]. While this characteristic of the MMP binding site has frustrated many attempts to develop selective systemic inhibitors directed at given MMPs [10,26], it makes it possible to develop unselective MMP inhibitors that could be applied topically to treat photoaging without triggering systemic side-effects [11]. Therefore, in this study we aim to develop and experimentally validate a virtual screening (VS) workflow for identifying broad-spectrum MMP inhibitors with the potential to delay the development of photoaging by topical administration. Moreover, considering the importance of natural bioactive compounds in the cosmetic industry nowadays, we have also applied our VS workflow to a database of naturally occurring compounds and identified potential non-selective MMP inhibitors of natural origin.

## 2. Results and Discussion

In order to obtain compounds that can simultaneously inhibit different MMP enzymes, we designed various VS workflows for MMP-1, -8, -9, -12 and -13 which targeted the conserved catalytic region in each of their binding sites. We then extracted the hit compounds that were obtained in two or more of these VSs and which, therefore, had the potential to bind non-specifically to more than one MMP. The approach described was applied to different compound libraries to: (a) validate the approach in silico (see Section 2.1); (b) validate the approach in vitro (see Section 2.2) and (c) use the approach to identify natural products that may be broad-spectrum MMP inhibitors (see Section 2.3).

### 2.1. VS Workflow Design and In Silico Validation

This section presents the steps of the various VS workflows that were performed for each MMP and their in silico validation.

#### 2.1.1. Random Forest Model

For each MMP, a random forest (RF) model based on circular fingerprints (FPs) was developed to rule out compounds with little probability of being active given their structural characteristics. Circular or Morgan FPs are based on the Morgan algorithm and they record the environment of each atom in the molecule up to a particular radius [27]. As hashed topological fingerprints, circular fingerprints do not refer to the presence or absence of a particular substructure specifically. Instead, they are built around each molecule so any molecule can produce a meaningful FP. Circular FPs were chosen in this step to enrich the compound library with actives for each MMP as they are one of the highest ranked FPs in terms of performance [27].

As a supervised machine learning algorithm is based on fingerprints, in these RF models, FP bits are related to bioactivity and the output probabilities are a function of the presence of important structural characteristics for the bioactivity of known actives. Therefore, the models should be capable of identifying molecules with structural characteristics similar to known actives, but with different overall structures, recognizing compounds that are most likely to be active. Because the RF model is not the last step of the VS workflows (see Table 1), compounds with a less-than-20% probability of being active were excluded. For this threshold, in a 5-fold cross-validation using actives and decoys, the RF models performed well (see Table 2 and the material and methods section for additional details). Thus, due to the low computational cost of FP calculations, these RF models enable compounds with a low probability of being active for the corresponding MMPs to be rapidly discarded.

#### 2.1.2. Protein-Ligand Docking

The compounds obtained from the RF step for MMP-8, -9, -12 and -13 were docked to the corresponding protein structures. The structures from the PDB [28] used for protein-ligand docking were 1ZVX [29], 4H2E [30], 1ROS [31] and 3KRY [32] for MMP-8, -9, -12 and -13, respectively. Protein-ligand docking allowed us to (1) discard compounds that were not expected to fit in the catalytic site of these MMPs and (2) generate docked poses or hypothetical binding modes of the compounds of our library in the catalytic sites of each enzyme.

#### 2.1.3. Pharmacophore

The next step in the workflow was a pharmacophoric filter. This step aimed to keep only the compounds that could perform the minimum required interactions to bind to the receptor [33]. For each MMP, a pharmacophore was built and validated using a set of actives and decoys (see Table 3 and the material and methods section). In the cases of MMP-8, -9, -12 and -13, the pharmacophores were built by docking a library of fragments to the binding site of the respective MMP crystal structures in order to probe the binding site and find the pharmacophoric sites that were most suitable for ligand binding [33]. In the case of MMP-1, a structure-based pharmacophore was not used; instead, a ligand-based pharmacophore was designed using compounds that were active towards MMP-1 [33]. The different pharmacophore hypotheses that were obtained were validated with a set of known active compounds for each MMP and a set of decoys obtained from the corresponding set of actives and the pharmacophore hypothesis with the best performance were selected in each validation for the screening of the compound library (see Table 3).

Once the hypotheses had been obtained and validated for each MMP, the successful compounds in the RF step were screened through the corresponding pharmacophore. In the case of MMP-8, -9, -12 and -13, the coordinates from the hypothetical binding modes generated in the docking step for each compound were used as the input for the pharmacophore screening so as to limit the results to only those conformations that should be able to fit in the binding site. In the case of MMP-1, conformations were generated for each compound and used as input for the pharmacophore screening.

#### 2.1.4. Electrostatic Similarity Analysis

In the case of MMP-8, -9, -12 and -13, the electrostatic potential of the docked poses remaining after the pharmacophore screening was compared to that of the co-crystallized ligands of the corresponding MMP. By selecting the compounds that have an electrostatic potential similar to that of a known active ligand, we aimed to keep the compounds that were most likely to match the electrostatic environment of the binding site of the corresponding MMP and bind to the corresponding binding site with greater affinity [33].

To perform these comparisons, the crystallized complexes of each MMP containing inhibitors with IC_50_ or K_i_ activity values between 1 and 100 nM were used as a reference. These crystallized complexes were superposed on the crystal structure that was used during the protein-ligand docking step in each case, so that the reference ligands were aligned to the docked poses obtained for each compound.

**Figure 1:**
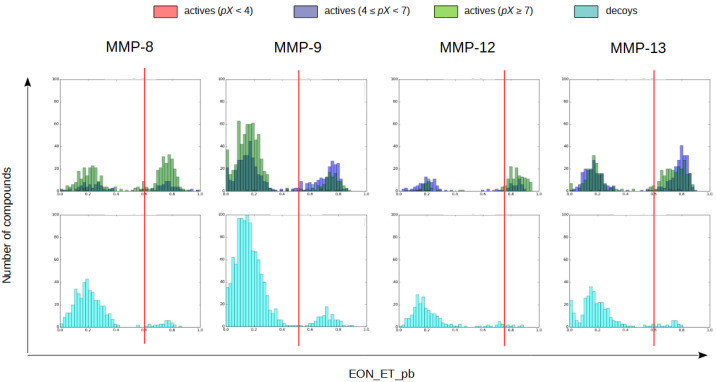
Histogram representations of the highest electrostatic Tanimoto (i.e., EON_ET_pb) values obtained in the comparison of the validation set with all queries. For each MMP, two histograms are shown: one for actives and one for decoys. In the actives histogram, actives with a *pX* lower than 4 are in red, actives with a *pX* between 4 and 7 are in blue and actives with a *pX* higher than 7 are in green. In the decoys histogram, decoys are in cyan. The EON_ET_pb cutoff is represented as a red line.

To validate in silico the use of these co-crystallized inhibitors as reference compounds for the electrostatic potential comparison, a validation set containing actives and decoys was prepared for each MMP and docked to the same protein structure used in the protein-ligand docking step. The electrostatic Tanimoto (i.e., EON_ET_pb) coefficients between the resulting docked poses and the set of experimental poses for the corresponding MMP were then calculated and only the docked pose with the highest EON_ET_pb value was kept regardless of the reference compound used for the comparison. After discarding all other docking poses, histograms of the electrostatic Tanimoto coefficients for the actives and the decoys in the validation set were plotted. The actives were separated into 3 groups depending on whether their bioactivity had a *pX* value lower than 4, between 4 and 7 or higher than 7. An EON_ET_pb cutoff was applied to each validation set on the basis of the distribution observed in the corresponding histogram (0.6, 0.5, 0.75 and 0.6 for MMP-8, -9, -12 and -13, respectively) so only the compounds whose electrostatic similarity was higher than that of known active compounds were kept in each case (see Figure 1).

### 2.2. In Vitro Validation

To validate this approach in vitro, a VS library was generated by collecting compounds from Specs [34] with molecular weights between 300 and 600 Da and generating a conformation for each compound with Omega [35]. This library was then subjected to the VS workflow. The number of compounds that surpassed the filters in each VS workflow can be found in Table 1. Despite the low threshold in the RF step, most of the compounds were discarded in each VS, indicating that their structures were unlikely to act as MMP inhibitors. Therefore, compounds that were not of interest were discarded at an early stage, thus reducing the computational cost of the VSs. After protein-ligand docking and the pharmacophoric filter many compounds remained, as sensitivity was prioritized in the validation of the pharmacophore models (see Table 3). The strictest/most demanding filter—the electrostatic similarity step—was applied last. It retained fewer than 100 compounds for each MMP and considerably reduced the number of hits.

**Figure 2:**
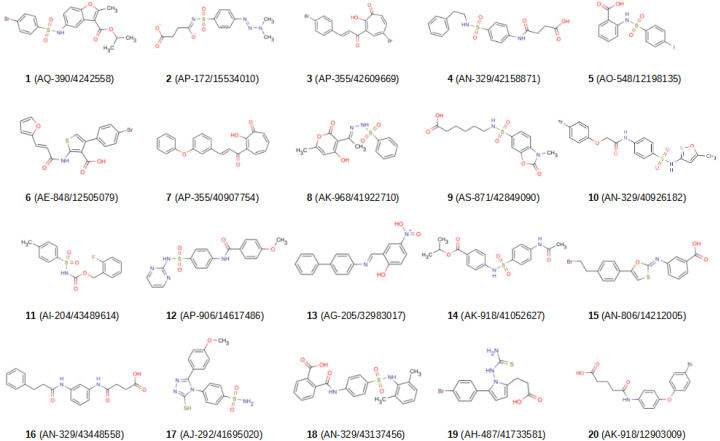
2D structures of the 20 hit compounds. Each compound is identified with its Specs ID number. MarvinSketch [38] was used to draw the structures.

Finally, we extracted the hits obtained in two or more VSs and clustered them together with the actives from the validation sets of all VSs steps using the HDBSCAN [36] algorithm in order to exclude hit compounds that were grouped in the same cluster as known active compounds. The clusters that contained only hit molecules were identified and 20 molecules belonging to different clusters were selected for activity tests. During that selection process, those molecules that had been hits in the highest number of VSs were prioritized relative to others from the same cluster. Finally, the 20 selected molecules were visually inspected to ensure that their structures were different. Figure 2 and Table S1 show the 2D structure, the canonical SMILES and the PAINS-REMOVER [37] prediction for the 20 selected molecules.

After the 20 hit compounds had been selected, they were purchased from Specs [34] and their activity for MMP-1, -8, -9, -12 and -13 was analyzed in vitro at a concentration of 100 μM. While the activities of compounds **2** (the only one of the 20 VS hits predicted as a PAIN [37]; see Table S1) and **13** could not be tested due to solubility and fluorescence problems, respectively, the other compounds (**1**, **3**–**12**, **14**–**20**) were able to inhibit several MMPs. Compounds **3**, **6**–**8** and **15** displayed the highest inhibitory activities, showing around 50% inhibition or more for all the MMPs tested (see Table 4). Next, the IC_50_ values were obtained for MMP-1, -8, -9, -12 and -13 of compounds **3**, **6**–**8** and **15** (see Table 5). Compound **3** proved to be the best broad-spectrum inhibitor of the five, its IC_50_ for MMP-1, -8, -9, -12 and -13 being 21, 23, 23, 24 and 35 μM, respectively.

**Figure 3:**
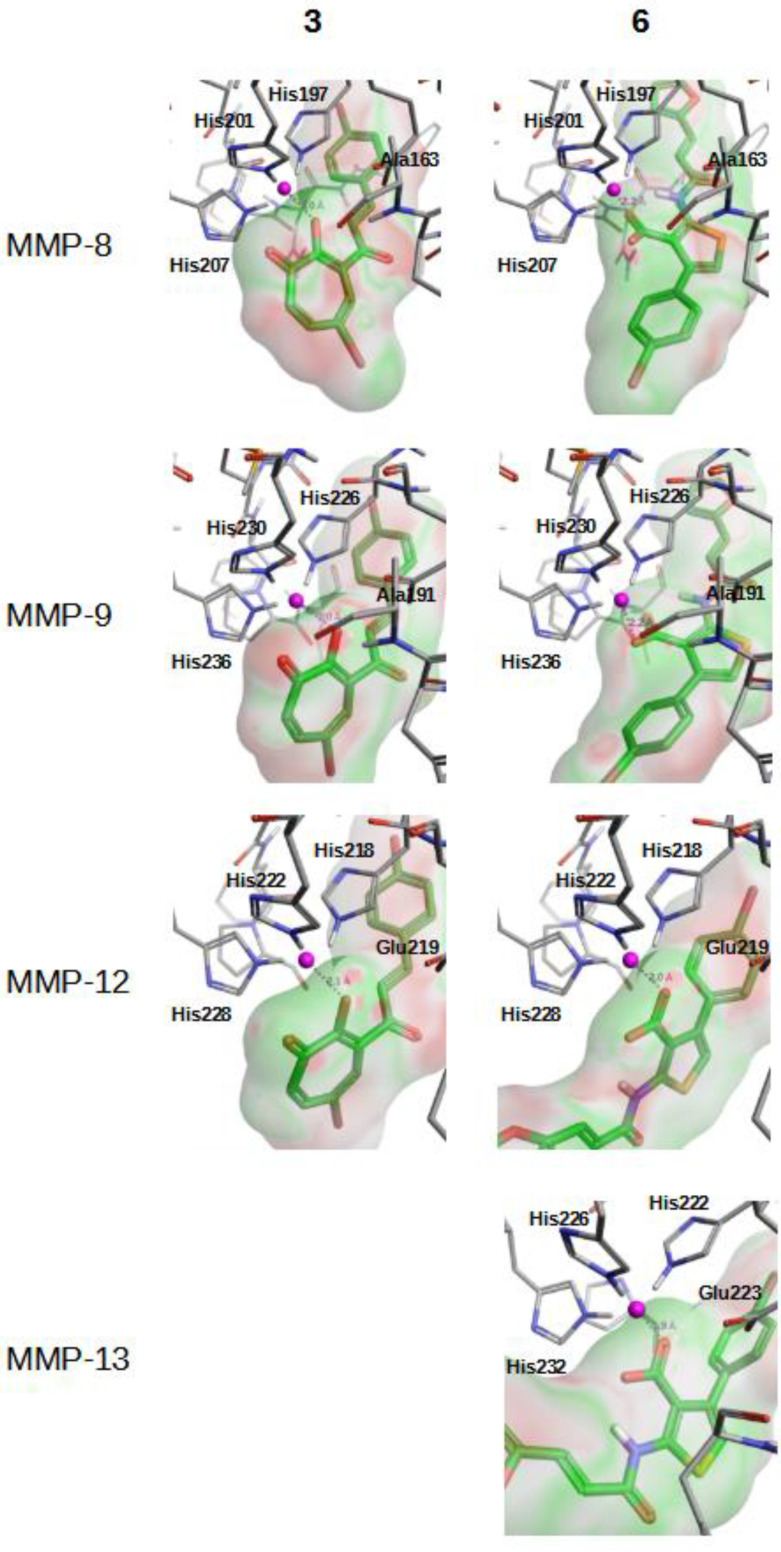
Best docking poses for hit compounds **3** and **6** at the Zn^2+^ binding site of MMP-8, MMP-9, MMP-12 and MMP-13. The docked poses for the **3**/MMP-13 pair is not shown because this was not predicted to be possible by the corresponding VS workflow.

Figure 3 and Figure S1 describe how compounds **3**, **6**–**8** and **15** bind at the Zn^2+^ binding site of MMP-8, MMP-9, MMP-12 and MMP-13 when this binding was predicted as possible by the corresponding VS workflow. As expected, ligand moieties either negatively charged (i.e., carboxylic acid in compounds **6** and **15**) or with lone electron pairs (i.e., hydroxyl group in compounds **3**, **7** and **8**) interact with the Zn^2+^ cation.

### 2.3. VS of Natural Products

**Figure 4:**
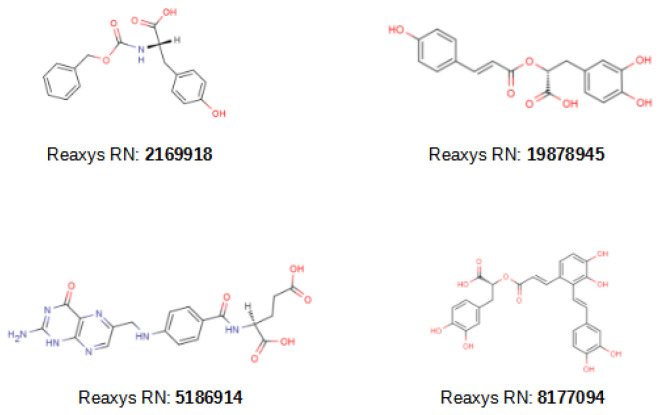
2D structures of the 4 natural hit compounds with either known MMP inhibitor bioactivity (i.e., **2169918** and **19878945**) or known application as dermatological agents for skin applications (i.e., **5186914** and **8177094**). Each compound is identified with its Reaxys register number (Reaxys RN). MarvinSketch [38] was used to draw the structures.

Having validated this methodology both in silico and in vitro, we proceeded to identify the natural compounds that can be used as broad-spectrum MMP inhibitors. For this purpose, a VS library was generated by collecting all the natural products in the Reaxys [39] database with a molecular weight between 300 and 600 Da and generating a conformation for each compound with Omega [35]. Then, the VS workflow was applied to this VS library and 183 compounds were obtained (see Table 1). Considering that 18 of the 20 compounds tested experimentally in the validation showed inhibitory activity for more than one MMP, many of these natural products were also expected to follow this pattern. Of these 183 natural products, 49 were hits by 3 or more VSs and were carefully inspected. Two of these 49 compounds have already been reported to inhibit MMP-2 and MMP-3, respectively (compounds with Reaxys [39] registry numbers **2169918** [40] and **19878945** [41]; see Figure 4), and we predicted that they could also inhibit other MMPs. Interestingly, another two of these 49 natural products were dermatological agents that are already being used for skin applications (compounds with Reaxys [39] registry numbers **5186914** [42,43,44,45,46,47,48,49,50,51,52] and **8177094** [53,54]; see Figure 4) and, if their MMP inhibitory activity was confirmed, this mechanism of action would explain their positive effects on skin ageing. Therefore, it is plausible that a significant portion of the remaining 47 natural compounds could also be useful for skin treatment (which will be investigated elsewhere).

**Figure 5:**
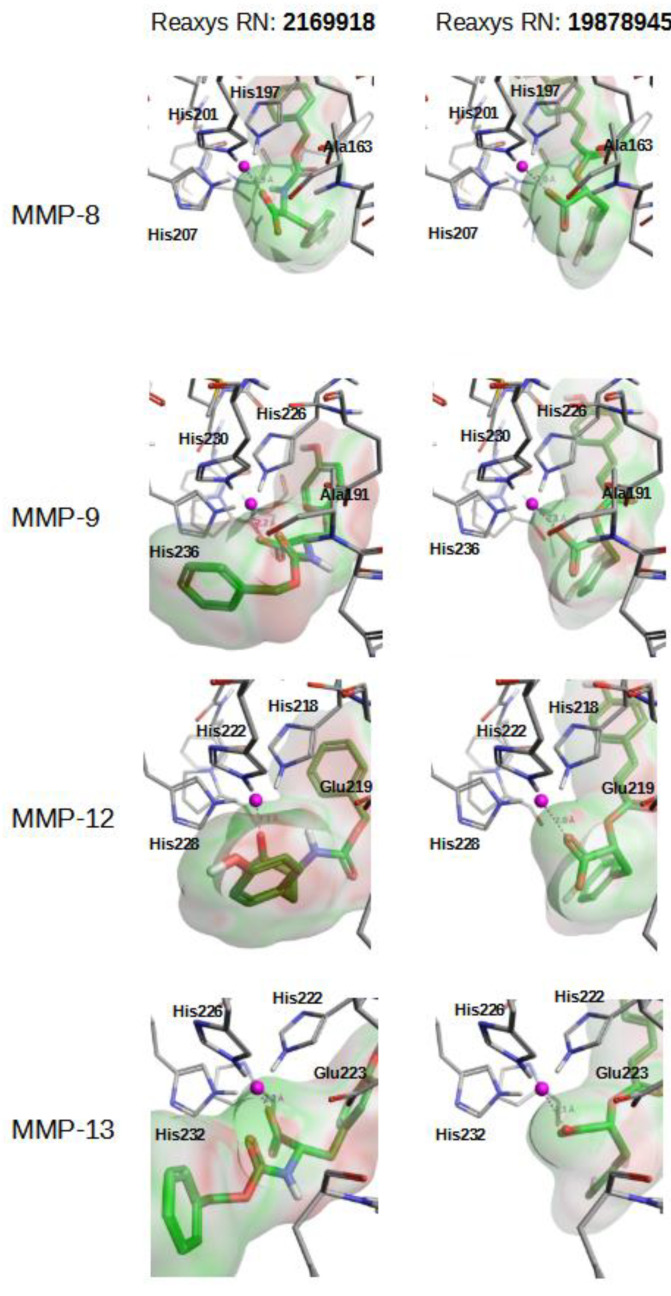
Best docking poses for natural compounds **2169918** and **19878945** at the Zn^2+^ binding site of MMP-8, MMP-9, MMP-12 and MMP-13.

Figure 5 and Figure S2 describe how compounds **2169918**, **19878945**, **5186914** and **8177094** bind at the Zn^2+^ binding site of MMP-8, MMP-9, MMP-12 and MMP-13. As expected, negatively charged ligand moieties (i.e., carboxylic acid in compounds **2169918**, **19878945**, **5186914** and **8177094**) interact with the Zn^2+^ cation.

## 3. Materials and Methods

### 3.1. RF Model

In order to prepare the molecules for the RF classifier model, the ChemAxon Standardizer [55] was used to generate their canonical representations. Morgan fingerprints of radius 2 were calculated with RDKit and were used as input descriptors to build the RF classifier to distinguish actives from decoys [56]. The RF model was built and validated with Scikit-learn and each MMP had a different set of actives and decoys [57]. The number of actives and decoys for each MMP were, respectively, 9796 and 9692 for MMP-1; 2931 and 2923 for MMP-8; 8439 and 8410 for MMP-9; 2610 and 2596 for MMP-12; and 6295 and 6284 for MMP-13. The actives were obtained from ChEMBL [58] and Reaxys [39] and are inhibitors of each human MMP whose bioactivity is in the 1–13 range for *pX*. Their activity was determined by measuring IC_50_ or K_i_. MW-based decoys were obtained from the ZINC [59] database using Decoyfinder [60]. Each model was built using 100 trees, their output classification probabilities were calibrated using Platt scaling [61] and they were validated by a 5-fold cross-validation in which the training and the test sets consisted of 80% and 20% of the compounds, respectively (see Table 2 for performance details).

### 3.2. Ligand Setup for Docking

Before docking, molecules were prepared with LigPrep [62] with default parameter values except in the following cases: (a) chiralities from input geometry were respected when generating stereoisomers; (b) Epik [63] was used for ionization and tautomerization; (c) metal binding states were added; (d) 7.0 was used as effective pH; and (e) 2.0 was used as pH tolerance for the structures generated.

### 3.3. Protein Preparation

After verifying the fitting of the coordinates of the residues in the binding site relative to their corresponding electron density map with VHELIBS [64], the crystal structures of MMP-8, -9, -12 and -13 (1ZVX [29] A chain, 4H2E [30] B chain, 1ROS [31] A chain and 3KRY [32] A chain, respectively) were obtained from the PDB [28] and prepared using Maestro’s Protein Preparation Wizard [65] and the following procedure: (a) original hydrogens were removed; (b) termini were capped; (c) ionization and tautomeric states of the ligand were generated with Epik [63]; (d) hydrogen bonds were assigned at pH 7 with PROPKA; (e) force field OPLS_2005 was used to minimize the structure at 0.30 Å; and (f) all water molecules were removed from the structure.

### 3.4. Grid Generation

The grid for protein-ligand docking was generated with Maestro [66] by using default parameter values and the following settings: (a) the grid center coordinates were (−2.0, 24.5, 8.3) for MMP-8, (26.0, 8.1, 50.4) for MMP-9, (52.4, 82.6, 6.8) for MMP-12 and (−11.2, −0.2, 2.1) for MMP-13; (b) the inner box size was (10, 10, 10) for all MMPs; and (c) the outer box size was (27.6, 27.6, 27.6) for MMP-8, (25, 25, 25) for MMP-9, (28.6, 28.6, 28.6) for MMP-12 and (28.7, 28.7, 28.7) for MMP-13.

### 3.5. Protein-Ligand Docking

Protein-ligand docking was performed with Glide [67] by using default parameter values except for the following settings: (a) SP precision; (b) the planarity of conjugated π groups was enhanced; (c) halogens were included as acceptors; (d) aromatic hydrogens were included as donors; (e) at most 10 poses were written out per ligand; and (f) 50 poses were included per ligand in post-docking minimization.

### 3.6. Pharmacophore Generation

To generate the pharmacophores for MMP-8, -9, -12 and -13, the Glide Fragment Library [68] was docked to the grid used for protein-ligand docking for each MMP with the default parameters and the following settings: (a) XP precision; (b) 50,000 initial poses were kept per ligand; (c) scoring cutoff was set to 500; (d) 1000 minimized poses were generated per ligand; and (e) expanded sampling was used. Next, the e-Pharmacophores [69,70] tool was used to group the fragments into 15 clusters and develop pharmacophore hypotheses with a maximum of 8 sites each. In the case of MMP-1, a ligand-based pharmacophore was generated with Phase [71] using a set of 916 actives of *pX* ≥ 7 and 57 inactives of *pX* ≤ 4 with the default parameters and the following settings: (a) up to 10 conformations were generated per ligand; (b) hypothesis should match at least 50% of actives; (c) number of features in the hypothesis: 3–5; and (d) preferred minimum number of features: 5. The resulting hypotheses were used to screen the validation library for each MMP with the default parameters and the following settings: (a) as many results as possible were kept and (b) in the case of MMP-8, -9, -12 and -13, the coordinates of the docked poses obtained during docking were used and, in the case of MMP-1, a maximum of 10 conformations were generated for each compound prior to screening. The respective number of actives and decoys used in the pharmacophore validation for each MMP were the following: 1395 and 1385 for MMP-1, 598 and 591 for MMP-8, 1800 and 1650 for MMP-9, 305 and 304 for MMP-12 and 938 and 906 for MMP-13. The results of the pharmacophore screening were analyzed and the hypothesis with the best performance was selected (see Table 3).

### 3.7. Electrostatic Similarity Analysis

EON software [72] compares the poses of two compounds by calculating Tanimoto coefficients associated to their electrostatic potentials (i.e., Poisson–Boltzmann electrostatics and the coulombic part of the Poisson–Boltzmann electrostatics), to their shape, or to a combination of both. The Poisson–Boltzmann electrostatics metric was used here to give an electrostatic Tanimoto value (i.e., EON_ET_pb) that was in the −⅓ to 1 range (where a value of 1 corresponds to identical electrostatic potential overlap and a negative value corresponds to the overlap of opposite charges between the two poses). The validation sets used in all electrostatic similarity comparisons consisted of the following numbers of actives and decoys, respectively, for each MMP: 600 and 599 for MMP-8, 1973 and 1964 for MMP-9, 310 and 308 for MMP-12 and 942 and 921 for MMP-13. The actives were obtained from Reaxys [39] and were inhibitors of human MMPs with a bioactivity between 1 and 13 for *pX*. Their activity was determined by measuring IC_50_ or K_i_. MW-based decoys were obtained from the ZINC [59] database using Decoyfinder [60]. Prior to the analysis, the docking of the validation set and the corresponding pharmacophore screening were performed following the procedure described above.

In order to determine which crystallized ligands to use as references for the electrostatic similarity analysis, the MMP crystal structures containing ligands with IC_50_ or K_i_ activity values between 1 and 100 nM (i.e., 6, 5, 18 and 19 crystal structures for MMP-8, -9, -12 and -13, respectively) were obtained and superposed on the crystal structure used for protein-ligand docking in each case (i.e., 1ZVX [29] for MMP-8, 4H2E [30] for MMP-9, 1ROS [31] for MMP-12 and 3KRY [32] for MMP-13). For each MMP, the validation set was then used to perform separate validations (i.e., one for each crystallized ligand in the superposed crystal structures) with the crystallized ligands as queries. In each validation, only the docked pose that presented the highest electrostatic Tanimoto with the corresponding query was kept for each library compound. After the rest of the docked poses had been discarded, histograms of the electrostatic Tanimoto coefficients for the actives and the decoys in the validation set were plotted, and the actives were separated into 3 groups depending on whether their *pX* value was lower than 4, between 4 and 7 or higher than 7 (see Figure S3). The crystallized ligands for which the electrostatic Tanimoto value distribution of the actives was very similar to that of the decoys were discarded, as no electrostatic Tanimoto value could be used as a cutoff to differentiate between the two groups in the validation set. The remaining crystallized actives for each MMP (i.e., ligands in crystal structures with the PDB [28] codes 1BZS [73], 1ZVX [29] and 3TT4 [74] for MMP-8; 2OVZ [75] and 2OW2 [75] for MMP-9; 1ROS [31], 2WO9 [76], 3EHX [77], 3TS4 [74], 4EFS [74], 4GR0 [78], 4GR3 [78] and 4GR8 [78] for MMP-12; and 3ELM [79] and 3TVC [74] for MMP-13) were selected as queries for the in silico validation of the electrostatic similarity analysis for the respective MMP (i.e., 3, 2, 8 and 2 crystallized ligands for MMP-8, -9, -12 and -13, respectively).

### 3.8. MMP Inhibition Assays

Pro-MMP-1, pro-MMP-8, pro-MMP-9 and pro-MMP-13 were purchased from Merck Millipore. Pro-MMP-12 was purchased from Bio-Techne. *p*-Aminophenylmercuric acetate (APMA) was from Sigma-Aldrich (Milan, Italy). Proenzymes were activated immediately prior to use with APMA 2 mM for 2 h at 37 °C for MMP-1, APMA 2 mM for 1 h at 37 °C for MMP-8, APMA 1 mM for 1 h at 37 °C for MMP-9, APMA 1 mM for 4 h at 37 °C for MMP-12 and APMA 1 mM for 30 min at 37 °C for MMP-13. For assay measurements, the purchased compound stock solutions (10 mM in DMSO) were further diluted for each MMP in the fluorometric assay buffer (FAB: Tris 50 mM, pH = 7.5, NaCl 150 mM, CaCl_2_ 10 mM, Brij 35 0.05% and DMSO 1%). Activated enzyme (final concentration 2.0 nM for MMP-1, 1.4 nM for MMP-8, 1.3 nM for MMP-9, 2.3 nM for MMP-12 and 0.3 nM for MMP-13) and inhibitor solutions were incubated in the assay buffer for 3 h at 25 °C. After the addition of 200 μM solution of the fluorogenic substrate Mca-Lys-Pro-Leu-Gly-Leu-Dap(Dnp)-Ala-Arg-NH2 (Merck Millipore) in DMSO (final concentration 2 μM), the hydrolysis was monitored every 15 s for 15 min and the increase in fluorescence (λ_ex_ = 325 nm, λ_em_ = 400 nm) was recorded using a Molecular Devices SpectraMax Gemini XPS plate reader. The assays were performed in triplicate in a total volume of 200 μL per well in 96-well microtiter plates (Corning, black, NBS). The MMP inhibition activity was expressed in relative fluorescent units (RFU). The percent of inhibition was calculated from control reactions without the inhibitor. The inhibitory effect of the compounds tested was routinely estimated at a concentration of 100 μM towards MMP-1, -8, -9, -12 and -13. Those compounds found to be active were tested at additional concentrations and IC_50_ was determined using at least five concentrations of the inhibitor, which caused an inhibition between 10% and 90%, using the formula *v_i_*/*v_o_* = 1/(1 + [I]/IC_50_), where *v_i_* is the initial velocity of substrate cleavage in the presence of the inhibitor at concentration [I] and *v_o_* is the initial velocity in the absence of the inhibitor. Results were analyzed using SoftMax Pro software and Origin 6.0 software.

## 4. Conclusions

In order to obtain potent unspecific MMP inhibitors, we developed a VS workflow designed to identify compounds that simultaneously target the Zn^2+^ binding region in different MMP enzymes. After validating the performance of this VS workflow in vitro with some selected VS hits obtained from the Specs library, we applied it to a subset of the Reaxys containing natural products with a molecular weight between 300 and 600 Da. Our predictions found that 49 of the resulting VS hits could inhibit at least 3 different MMPs and, interestingly, that 2 of these 49 compounds are already used for skin care applications and another 2 are known MMP inhibitors. Consequently, our work paves the way for the discovery of new non-selective MMP inhibitors of natural origin that could be used as bioactive cosmetic compounds for the treatment of photoaging. Therefore, the characterization of natural extracts containing any of these 49 compounds merits further attention, and current work in this regard is underway. Finally, given that non-selective MMP inhibitors have also been used to inhibit nematode-specific metalloproteases [80,81], it is possible that our VS hits (either those from the Specs library or those from natural origin) may also be useful as lead molecules to design more potent drugs to treat parasitic nematode infection in humans and animals.

## Figures and Tables

**Table 1:** VS workflow filters and number of compounds that surpassed each VS filter for each MMP.

	Specs					Reaxys NP				
	MMP-1	MMP-8	MMP-9	MMP-12	MMP-13	MMP-1	MMP-8	MMP-9	MMP-12	MMP-13
Initial library	45,711					105,050				
Random forest	1344	4576	3334	3819	1584	5878	5811	11,070	17,684	2991
Protein-ligand docking	-	3775	2760	3001	1314	-	2401	4970	6896	1285
Pharmacophore	1064	2762	785	1055	454	3358	958	905	987	209
Electrostatic similarity analysis	-	79	60	32	27	-	118	314	102	70
Hits of 2 or more VSs	54					183				

**Table 2:** Statistical parameters of the RF model validation for each MMP. The values correspond to the means of the 5-fold cross-validation for each parameter.

MMP	Sensitivity	Specificity	Precision	Fall-Out	False Negative Rate	False Discovery Rate	Accuracy	F1 Score	Matthews Correlation Coefficient
MMP-1	0.99	0.98	0.98	0.02	0.01	0.02	0.99	0.99	0.97
MMP-8	0.99	0.97	0.97	0.03	0.01	0.03	0.98	0.98	0.96
MMP-9	0.99	0.98	0.98	0.02	0.01	0.02	0.98	0.98	0.97
MMP-12	0.99	0.98	0.98	0.02	0.01	0.02	0.98	0.98	0.96
MMP-13	0.99	0.97	0.97	0.03	0.01	0.03	0.98	0.98	0.96

**Table 3:** Pharmacophore hypotheses selected for each MMP and statistical parameters obtained in their validation. The letters “A”, “D”, “R” and “N” stand for acceptor, donor, aromatic and negative pharmacophoric features, respectively, and the symbols “+” and “−” indicate whether they match the pharmacophoric site or not.

MMP	Pharmacophore Hypothesis	Sensitivity	Specificity	Precision	Fall-Out	False Negative Rate	False Discovery Rate	Accuracy	F1 Score	Matthews Correlation Coefficient
MMP-1	A (+) A (+) R (+)	0.89	0.33	0.57	0.67	0.11	0.43	0.61	0.70	0.26
MMP-8	A (+) A (−) D (−) D (−) D (−) D (−) R (+)	0.88	0.55	0.66	0.45	0.12	0.34	0.72	0.76	0.46
MMP-9	A (−) A(+) D (−) D (−) H (−) N (+) R (−) R (−)	0.67	0.86	0.84	0.14	0.33	0.16	0.76	0.74	0.54
MMP-12	A (−) D (−) D (−) N (+) R (+) R (−)	0.82	0.75	0.76	0.25	0.18	0.24	0.78	0.79	0.57
MMP-13	A (−) D (−) D (−) D (−) N (+) R (−) R (+)	0.82	0.74	0.76	0.26	0.18	0.24	0.78	0.79	0.56

**Table 4:** MMP inhibitory activity of the compounds tested (inhibition % at 100 μM) ^a^.

Compound	MMP-1	MMP-8	MMP-9	MMP-12	MMP-13
**1**	10.5%	19.6%	18.2%	16.1%	21.3%
**2** ^b^	ND	ND	ND	ND	ND
**3**	84.2%	80.9%	80.1%	79.7%	69.5%
**4**	17.5%	19.8%	24.8%	17.8%	17.8%
**5**	25.2%	27.8%	32.0%	26.3%	22.4%
**6**	71.2%	68.5%	78.1%	70.4%	77.8%
**7**	74.0%	71.1%	72.7%	73.5%	60.0%
**8**	50.9%	53.1%	59.3%	56.5%	48.8%
**9**	17.6%	8.7%	14.9%	18.9%	16.8%
**10**	19.1%	21.7%	25.6%	31.5%	19.4%
**11**	5.5%	6.0%	27.8%	19.3%	18.8%
**12**	23.7%	10.8%	28.4%	31.2%	20.9%
**13 ^c^**	ND	ND	ND	ND	ND
**14**	18.9%	25.6%	26.1%	30.4%	31.2%
**15**	50.3%	66.5%	60.2%	51.8%	71.3%
**16**	15.3%	5.6%	20.5%	58.4%	20.0%
**17**	20.4%	22.6%	76.8%	35.5%	27.3%
**18**	10.0%	20.4%	32.3%	52.6%	28.4%
**19**	32.0%	30.3%	69.7%	32.6%	37.8%
**20**	6.4%	29.2%	27.2%	32.2%	26.9%

^a^ Percent inhibition of MMPs observed at 100 µM concentration of the test compounds. Assays were performed in triplicate. ^b^ Insoluble in DMSO. ^c^ Fluorescent at 430 nm. ND refers to “not determined”.

**Table 5:** MMP inhibitory activity ^a^ of the compounds tested (IC_50_ values in µM).

Compound	MMP-1	MMP-8	MMP-9	MMP-12	MMP-13
**3**	21 ± 2	23 ± 2	23 ± 1	24 ± 1	35 ± 3
**6**	32 ± 4	31 ± 5	26 ± 2	33 ± 5	33 ± 4
**7**	41 ± 2	41 ± 5	31 ± 1	30 ± 2	62 ± 4
**8**	92 ± 9	103 ± 17	80 ± 4	107 ± 9	108 ± 10
**15** ^b^	70 ± 9	77 ± 10	47 ± 6	111 ± 1	46 ± 7

^a^ Assays were run in triplicate. The final values given here are the mean ± SD of three independent experiments. ^b^ Low solubility in buffer.

## Data Availability

Not applicable.

## Supplementary Materials

The following are available online. Table S1: Canonical SMILES and PAINS-REMOVER (https://www.cbligand.org/PAINS/) results for the 20 compounds selected for in vitro validation. Figure S1: Best docking poses (when possible) for hit compounds **7**, **8** and **15** at the Zn^2+^ binding site of MMP-8, MMP-9, MMP-12 and MMP-13. Figure S2: Best docking poses for natural compounds **5186914** and **8177094** at the Zn^2+^ binding site of MMP-8, MMP-9, MMP-12 and MMP-13. Figure S3: Histogram representations of the highest electrostatic Tanimoto (i.e., EON_ET_pb) values obtained when comparing the validation set to each query. Panels A, B, C and D show the validations for each of the queries for MMP-8, -9, -12 and -13, respectively. For each query, two histograms are shown: one corresponding to the actives and one corresponding to the decoys. In the actives histogram, actives with a *pX* lower than 4 are in red, actives with a *pX* between 4 and 7 are in blue, and actives with a *pX* higher than 7 are in green. In the decoys histogram, decoys are in cyan.

## Sample Availability

All the mentioned compounds can be bought from either Specs or other chemical suppliers.
