# Supplementary material for: Identification of Broad-Spectrum MMP Inhibitors by Virtual Screening
Source: Molecules. 2021 Jul 28;26(15):4553. doi: 10.3390/molecules26154553 (PMC8347235; doi:10.3390/molecules26154553)
Supplement: Supplementary file 1 [file molecules-26-04553-s001.zip › molecules-1183003-supplementary.pdf]

**Supplementary materials for**

# *Identification of broad-spectrum MMP inhibitors by virtual screening*

Aleix Gimeno,[1] Doretta Cuffaro,[2] Elisa Nuti,[2] María José Ojeda-Montes,[1] Raúl Beltrán-Debón,[1] Miquel Mulero,[1] Armando Rossello,[2] Gerard Pujadas,[1*] Santiago Garcia-Vallvé[1*]

[1] Research group in Cheminformatics & Nutrition, Departament de Bioquímica i Biotecnologia, Universitat Rovira i Virgili, Campus de Sescelades, 43007 Tarragona, Catalonia, Spain

[2] Department of Pharmacy, University of Pisa, Via Bonanno 6, 56126 Pisa, Italy

* Correspondence to gerard.pujadas@gmail.com (GP); santi.garcia-vallve@urv.cat (SGV)

**Table S1.** Canonical SMILES and PAINS-REMOVER (https://www.cbligand.org/PAINS/) results for the 20 compounds selected for *in vitro* validation.

| Molecule ID | Canonical SMILES | Classified as a PAINS by PAINS-REMOVER |
|---|---|---|
| **1** | CC(OC(=O)c1c(C)oc2c1cc(cc2)NS(=O)(=O)c1ccc(cc1)Br)C | No |
| **2** | CN(/N=N/c1ccc(cc1)S(=O)(=O)/N=C(/CCC(=O)[O-])\[O-])C | Yes |
| **3** | Brc1ccc(cc1)/C=C/C(=O)c1cc(Br)ccc(=O)c1O | No |
| **4** | O=C(Nc1ccc(cc1)S(=O)(=O)NCCc1ccccc1)CCC(=O)O | No |
| **5** | Ic1ccc(cc1)S(=O)(=O)Nc1ccccc1C(=O)O | No |
| **6** | O=C(Nc1scc(c1C(=O)O)c1ccc(cc1)Br)/C=C/c1ccco1 | No |
| **7** | O=C(c1ccccc(=O)c1O)/C=C/c1cccc(c1)Oc1ccccc1 | No |
| **8** | Cc1cc(O)c(c(=O)o1)/C(=N/NS(=O)(=O)c1ccccc1)/C | No |
| **9** | OC(=O)CCCCCNS(=O)(=O)c1ccc2c(c1)oc(=O)n2C | No |
| **10** | O=C(Nc1ccc(cc1)S(=O)(=O)Nc1noc(c1)C)COc1ccc(cc1)Br | No |
| **11** | O=C(NS(=O)(=O)c1ccc(cc1)C)OCc1ccccc1F | No |
| **12** | COc1ccc(cc1)C(=O)Nc1ccc(cc1)S(=O)(=O)Nc1ncccn1 | No |
| **13** | Oc1ccc(cc1/C=N/c1ccc(cc1)c1ccccc1)[N+](=O)O | No |
| **14** | CC(=O)Nc1ccc(cc1)S(=O)(=O)Nc1ccc(cc1)C(=O)OC(C)C | No |
| **15** | BrCCc1ccc(cc1)c1cs/c(=N\c2cccc(c2)C(=O)O)/o1 | No |
| **16** | O=C(Nc1cccc(c1)NC(=O)CCC(=O)O)CCc1ccccc1 | No |
| **17** | COc1ccc(cc1)c1nnc(n1c1ccc(cc1)S(=O)(=O)N)S | No |
| **18** | OC(=O)c1ccccc1C(=O)Nc1ccc(cc1)S(=O)(=O)Nc1c(C)cccc1C | No |
| **19** | OC(=O)CCc1ccc(n1NC(=S)N)c1ccc(cc1)Br | No |
| **20** | O=C(Nc1ccc(cc1)Oc1ccc(cc1)Br)CCCC(=O)O | No |

**Figure S1.** Best docking poses for hit compounds **7**, **8** and **15** at the $Zn^{2+}$ binding site of MMP-8, MMP-9, MMP-12 and MMP-13. Docked poses for **15**/MMP-8, **7**/MMP-12 and **8**/MMP-13 pairs are not shown because these compounds/target pairs were not predicted to be possible by the virtual screening workflows.

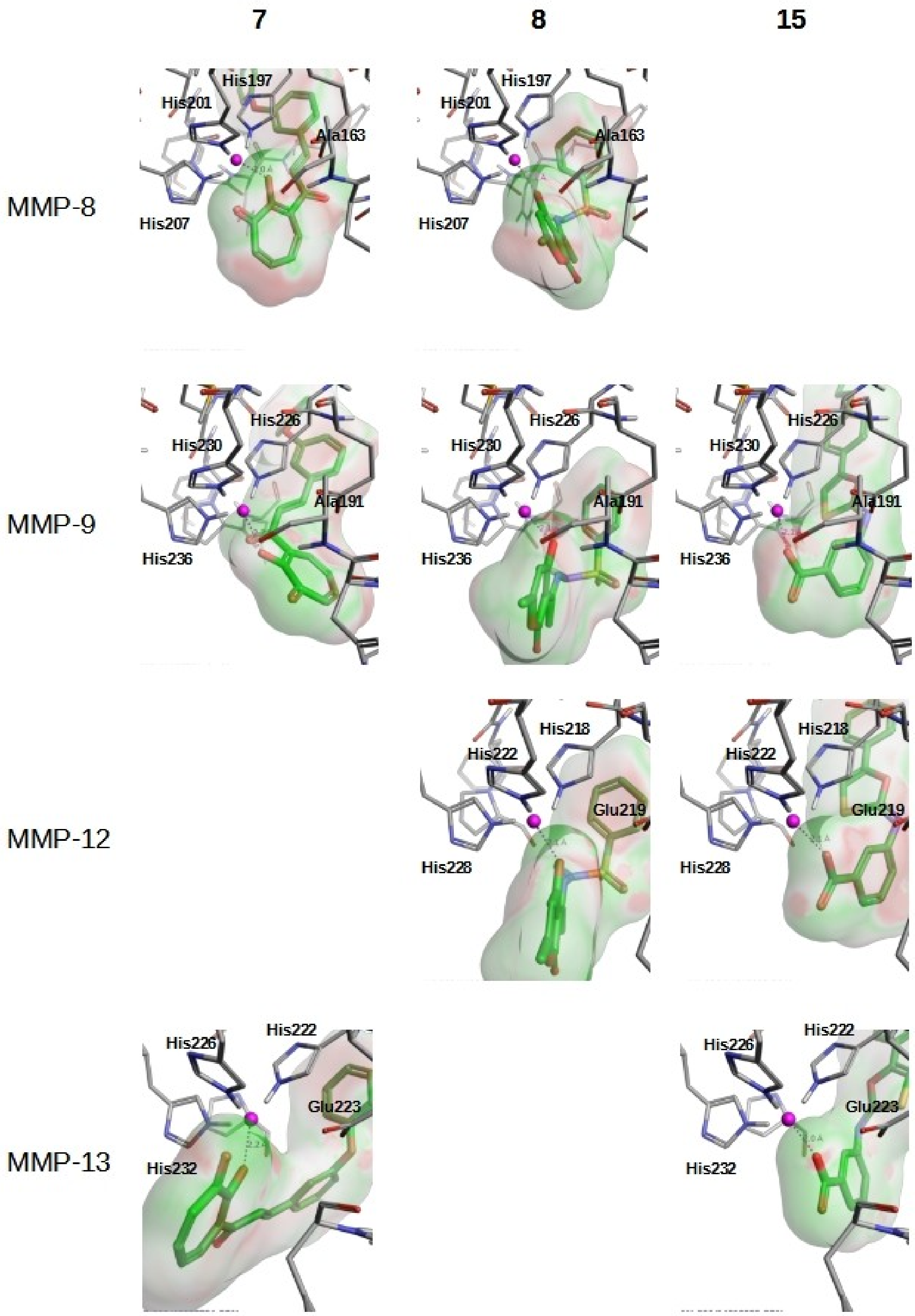

**Figure S2.** Best docking poses for natural compounds **5186914** and **8177094** at the $Zn^{2+}$ binding site of MMP-8, MMP-9, MMP-12 and MMP-13.

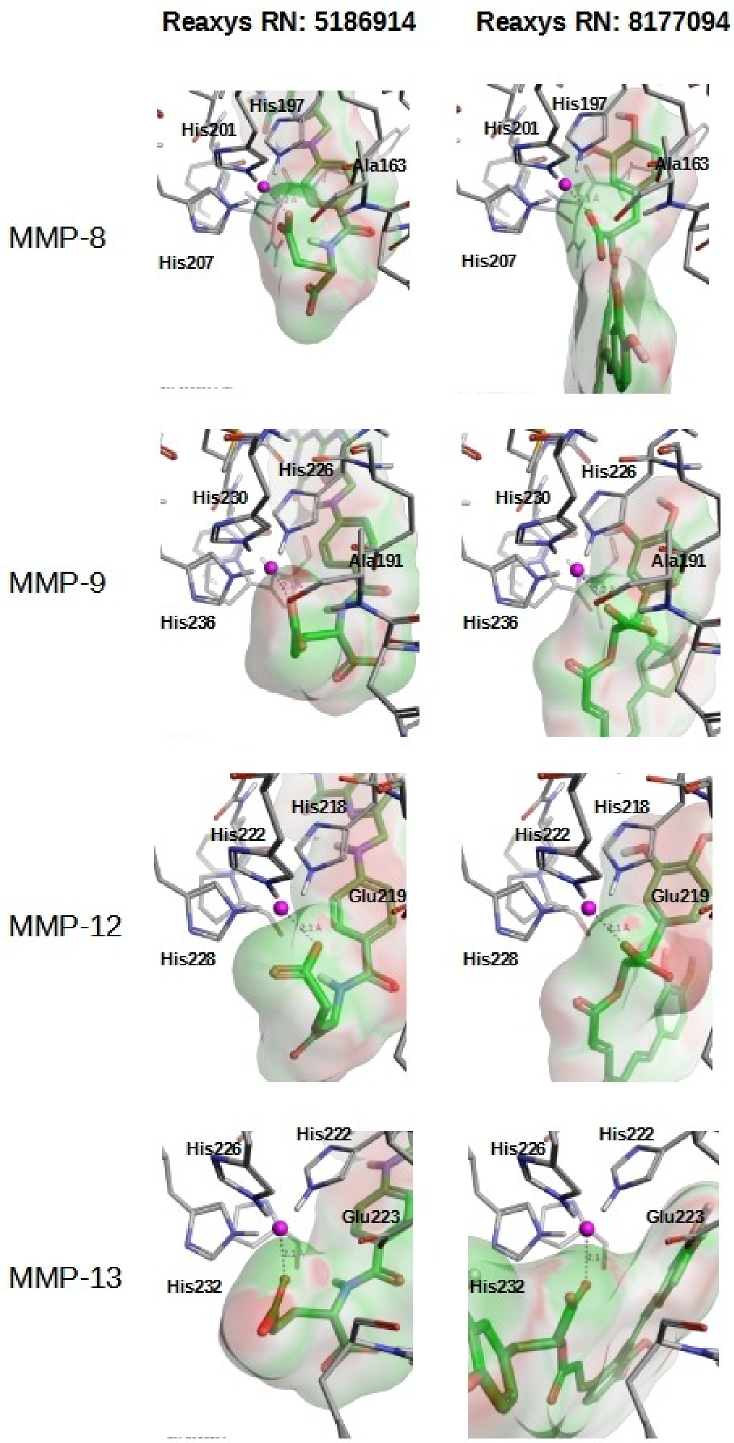

**Figure S3.** Histogram representations of the highest electrostatic Tanimoto (*i.e.* EON_ET_pb) values obtained when comparing the validation set to each query. Panels A, B, C and D show the validations for each of the queries for MMP-8, -9, -12 and -13, respectively. For each query, two histograms are shown: one corresponding to the actives and one corresponding to the decoys. In the actives histogram, actives with a pX lower than 4 are in red, actives with a pX between 4 and 7 are in blue, and actives with a pX higher than 7 are in green. In the decoys histogram, decoys are in cyan.

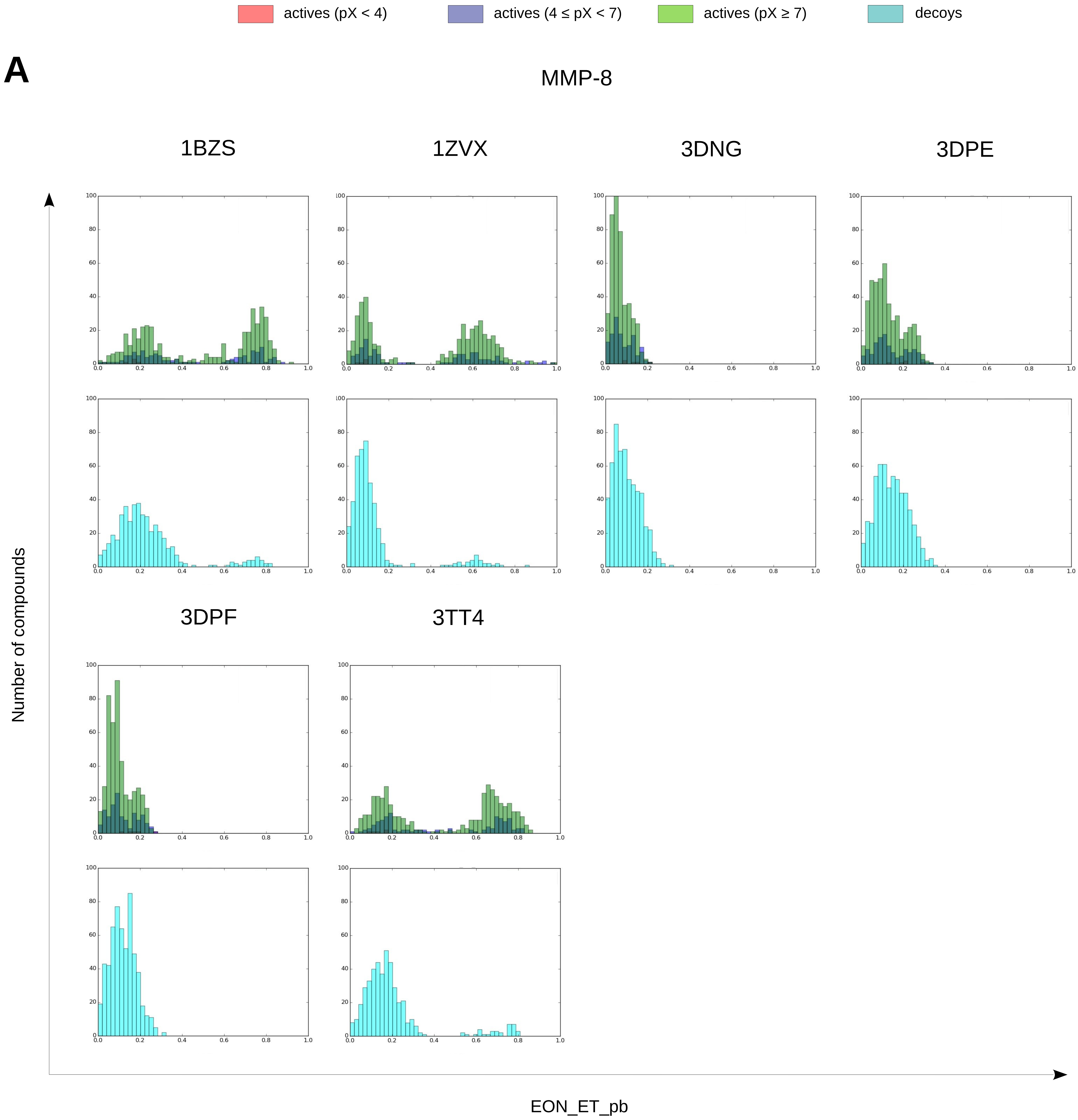

actives (pX < 4)
actives (4 ≤ pX < 7)
actives (pX ≥ 7)
decoys
A
MMP-8
1BZS
1ZVX
3DNG
3DPE
3DPF
3TT4
Number of compounds
EON_ET_pb

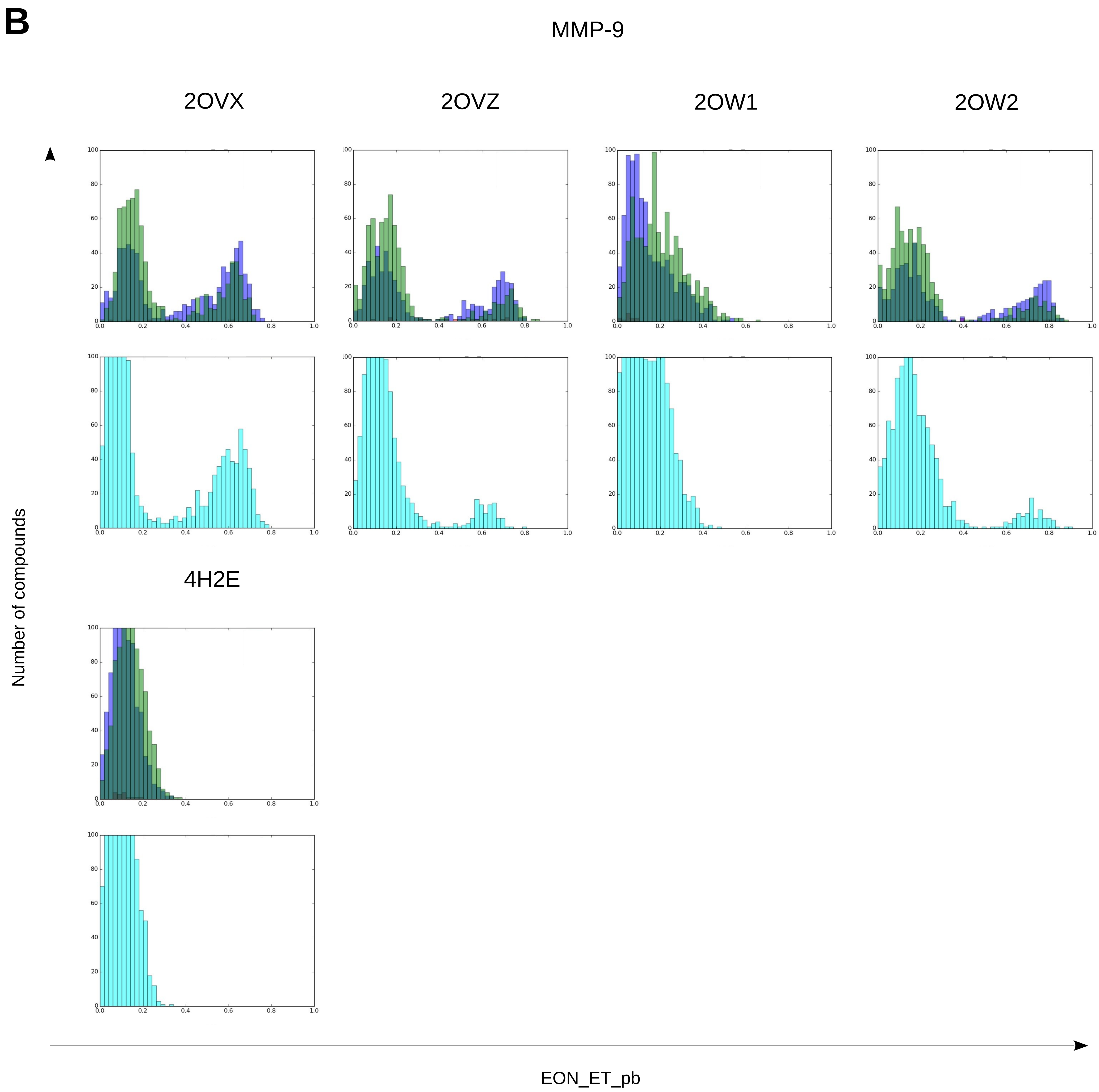

B
MMP-9
2OVX
2OVZ
2OW1
2OW2
4H2E
Number of compounds
EON_ET_pb

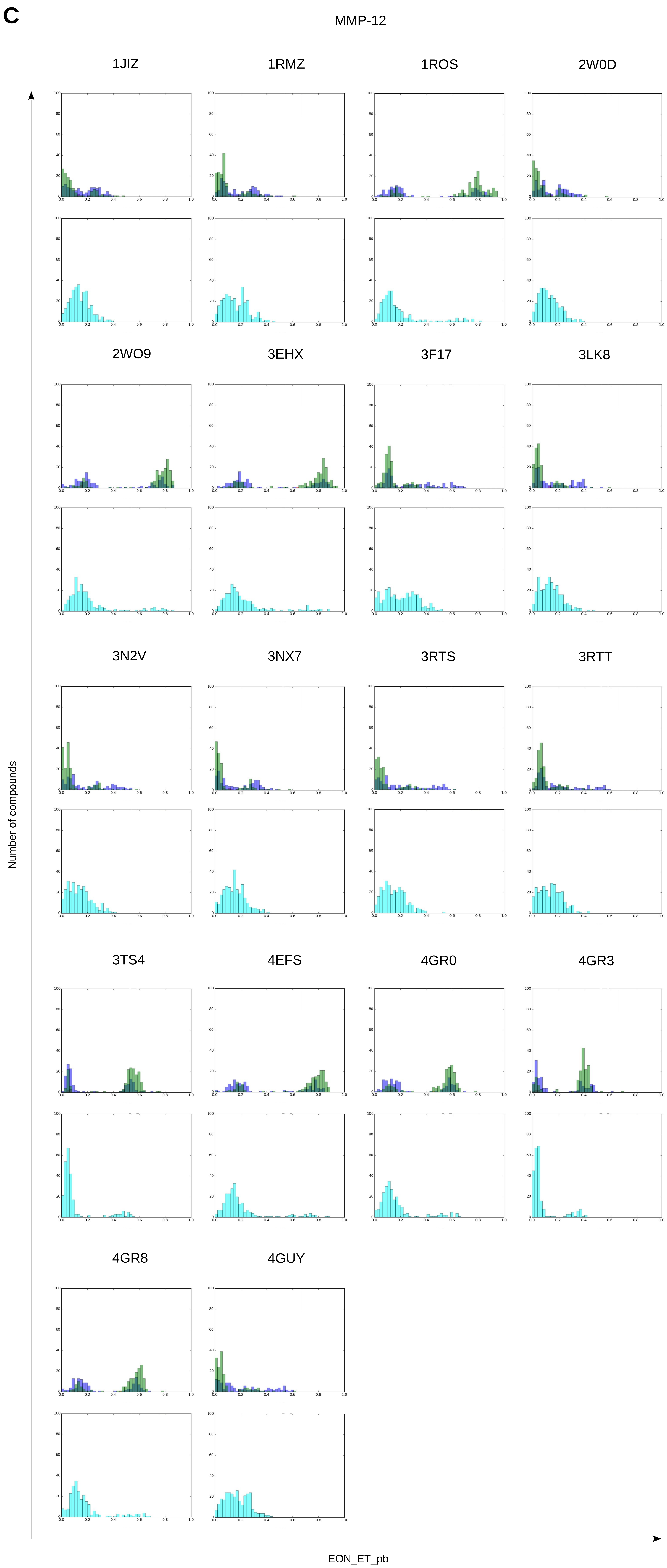
C
MMP-12
1JIZ
1RMZ
1ROS
2W0D
2WO9
3EHX
3F17
3LK8
3N2V
3NX7
3RTS
3RTT
3TS4
4EFS
4GR0
4GR3
4GR8
4GUY
Number of compounds
EON_ET_pb

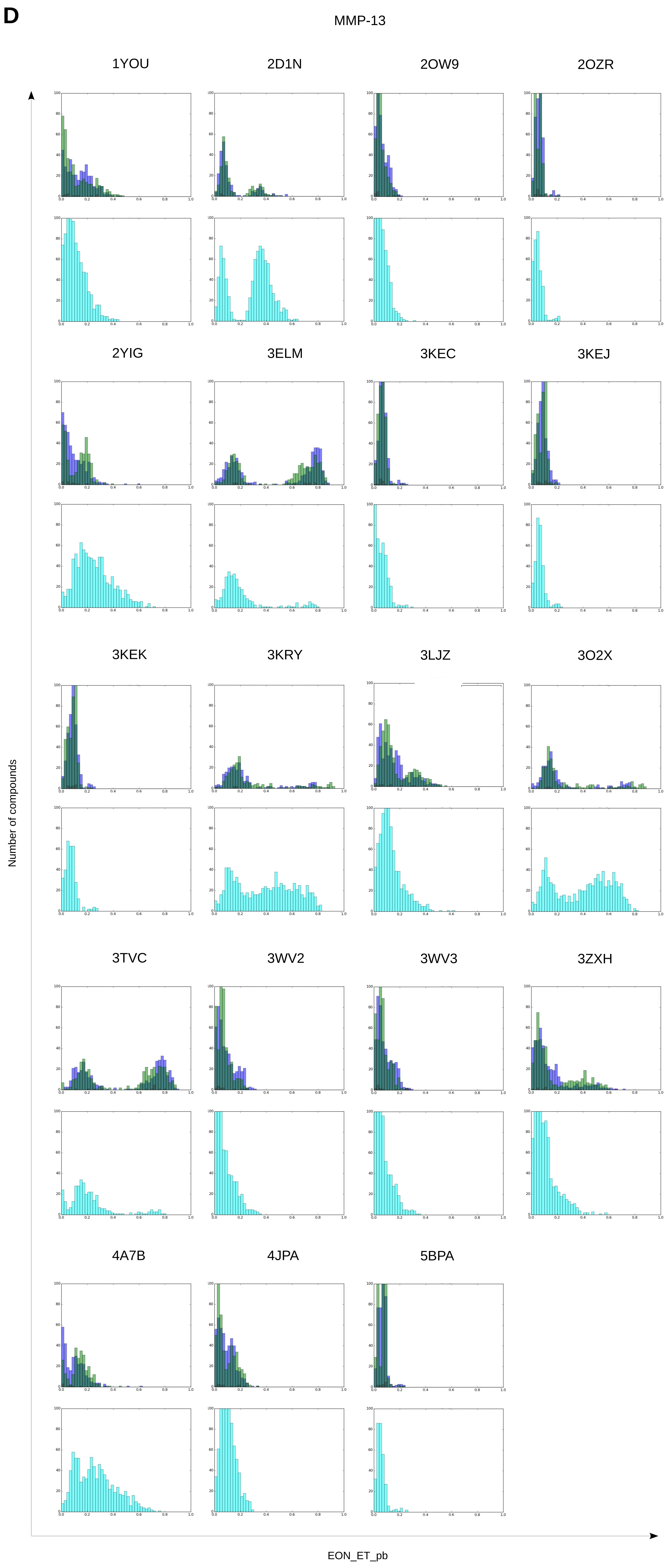
D
MMP-13
1YOU
2D1N
2OW9
2OZR
2YIG
3ELM
3KEC
3KEJ
3KEK
3KRY
3LJZ
3O2X
3TVC
3WV2
3WV3
3ZXH
4A7B
4JPA
5BPA
Number of compounds
EON_ET_pb
